# Hybridization of mouse lemurs: different patterns under different ecological conditions

**DOI:** 10.1186/1471-2148-11-297

**Published:** 2011-10-11

**Authors:** Andreas Hapke, Mark Gligor, S Jacques Rakotondranary, David Rosenkranz, Oliver Zupke

**Affiliations:** 1 Institut für Anthropologie, Johannes-Gutenberg-Universität Mainz, Colonel-Kleinmann-Weg 2, 55099 Mainz, Germany; 2 Forschungszentrum Jülich GmbH, Projektträger Jülich, BIO1, 52425 Jülich, Germany; 3 Department of Animal Ecology and Conservation, University of Hamburg, Biozentrum Grindel, Martin-Luther-King Platz 3, 20146 Hamburg, Germany; 4 III. Medizinische Klinik und Poliklinik, Johannes-Gutenberg-Universität Mainz, Langenbeckstr. 1, 55131 Mainz, Germany

## Abstract

**Background:**

Several mechanistic models aim to explain the diversification of the multitude of endemic species on Madagascar. The island's biogeographic history probably offered numerous opportunities for secondary contact and subsequent hybridization. Existing diversification models do not consider a possible role of these processes. One key question for a better understanding of their potential importance is how they are influenced by different environmental settings. Here, we characterized a contact zone between two species of mouse lemurs, *Microcebus griseorufus *and *M. murinus*, in dry spiny bush and mesic gallery forest that border each other sharply without intermediate habitats between them. We performed population genetic analyses based on mtDNA sequences and nine nuclear microsatellites and compared the results to a known hybrid zone of the same species in a nearby wide gradient from dry spiny bush over transitional forest to humid littoral forest.

**Results:**

In the spiny-gallery system, *Microcebus griseorufus *is restricted to the spiny bush; *Microcebus murinus *occurs in gallery forest and locally invades the dryer habitat of its congener. We found evidence for bidirectional introgressive hybridization, which is closely linked to increased spatial overlap within the spiny bush. Within 159 individuals, we observed 18 hybrids with mitochondrial haplotypes of both species. Analyses of simulated microsatellite data indicate that we identified hybrids with great accuracy and that we probably underestimated their true number. We discuss short-term climatic fluctuations as potential trigger for the dynamic of invasion and subsequent hybridization. In the gradient hybrid zone in turn, long-term aridification could have favored unidirectional nuclear introgression from *Microcebus griseorufus *into *M. murinus *in transitional forest.

**Conclusions:**

Madagascar's southeastern transitional zone harbors two very different hybrid zones of mouse lemurs in different environmental settings. This sheds light on the multitude of opportunities for the formation of hybrid zones and indicates an important influence of environmental factors on secondary contact and hybridization. Our findings suggest that hybridization could enhance the adaptability of mouse lemurs without necessarily leading to a loss of distinctiveness. They point to a potential role of hybridization in Madagascar's diversification history that requires further investigation.

## Background

There is increasing evidence that natural hybridization between animal species occurs more frequently than previously appreciated [1,2]. Moreover, the use of genetic techniques has facilitated the detection of many cases of introgressive hybridization between animals [3]. Hybridization can constitute a threat for biodiversity when rare endemic species come into contact with widespread invaders and are hybridized out of existence [4,5], but there are also examples of hybridizing species that maintain distinctiveness in the face of interspecific gene flow (e.g. [6,7]). Introgressive hybridization can even allow for the transfer of beneficial adaptations between species, facilitate rapid adaptation to changing environmental conditions and thus play an important role for diversification [2,3,8]. According to different models, environmental factors can influence hybrid zones to various degrees. Tension zones are maintained by a balance of immigration of the parental species and endogenous selection against hybrids, which is independent of environmental factors [9]. In other kinds of hybrid zones, environmental selection in mosaics or gradients of different habitats influences hybridization [1,10,11]. According to the tension zone and mosaic zone models, hybrids are generally less fit than the parental species [9,11]. According to the bounded hybrid superiority model [10], they are more fit than the parental species in ecotonal habitats. Under the evolutionary novelty model [1], endogenous and environmental selection act in concert, and certain hybrid genotypes can be as fit as or more fit than the parental species in ecotonal and parental habitats. Temporal change of environmental conditions can also influence hybridization. Prominent examples for hybrid superiority due to climatic fluctuations are Darwin's finches on Daphne Major Island [12].

Madagascar has an extremely high level of endemism both at the species level and at higher taxonomic levels and is among the world's eight hottest biodiversity hotspots [13]. Due to its long isolation from other landmasses, the island harbors many endemic radiations that gave rise to numerous microendemic species with very restricted ranges [14-16]. In various groups of organisms, there are also macroendemic species with larger ranges that overlap with those of their microendemic congeners. Examples are leaf chameleons [17,18], cophyline frogs [17,19], tufted-tailed rats [20] and mouse lemurs [21]. Patterns of microendemism in Madagascar are just beginning to emerge in the course of a recent and ongoing wave of species detections [14,22]. In recent years, a variety of models have been proposed that aim to explain the evolution of this diversity of microendemics [15,19,23,24]. Most of these models focus on allopatric or vicariant speciation. Consequently, a potential role of hybridization for the diversification of Malagasy endemics has so far widely been neglected although the present biogeography offers numerous opportunities for the formation of hybrid zones along environmental gradients ([16] but see [24]). There is a controversial discussion about a potential role of Pleistocene climatic changes for the evolution of microendemics in Madagascar [15,18,24]. At least it is clear that Madagascar's current biogeography arose after dramatic changes in the course of climatic fluctuations, which are best documented for the Holocene [25]. Most endemic organisms must have undergone range shifts, retractions and expansions in the course of climatic fluctuations [15]. The study of microevolutionary processes at species boundaries is thus important for a better understanding of how species adapted to changing environmental conditions and how the diversity of microendemics was maintained throughout the vicissitudes of the Pleistocene. It appears probable that there were even more opportunities for the formation of hybrid zones in the dynamic past of Madagascar's biogeography than is evident from current patterns. At present, there is a belt of humid forest in eastern Madagascar, seasonally dry deciduous forest in western Madagascar and dry spiny bush in the South. Dry western and humid eastern formations are largely separated from each other by mostly forestless central highlands. Dry-humid ecotones are restricted to the North and the South, but there is evidence for the existence of forest corridors that connected western dry and eastern humid forest through the central highlands during the Holocene [23,26]. Heckman et al. [27] discuss secondary contact through such corridors and hybridization as potential alternative explanation to incomplete lineage sorting for phylogeographic patterns in western and eastern mouse lemurs.

There is an ongoing discussion about the question how many species of mouse lemurs should be distinguished (e.g. [28,29]). Weisrock et al. [29] delineated 16 population-level lineages based on sequence data from two mitochondrial and four nuclear loci from a large sample of mouse lemurs comprising localities all across Madagascar. According to the authors [29], it depends on the species concept applied how many of the 16 lineages deserve species rank. The currently recognized species *Microcebus murinus *comprises several of these lineages. Weisrock et al. [29] did not formally subdivide *Microcebus murinus *into different species. In the study presented here, we use the name "*Microcebus murinus*" in the sense of "*M. murinus *sensu latu" in Weisrock et al. [29].

**Figure 1:**
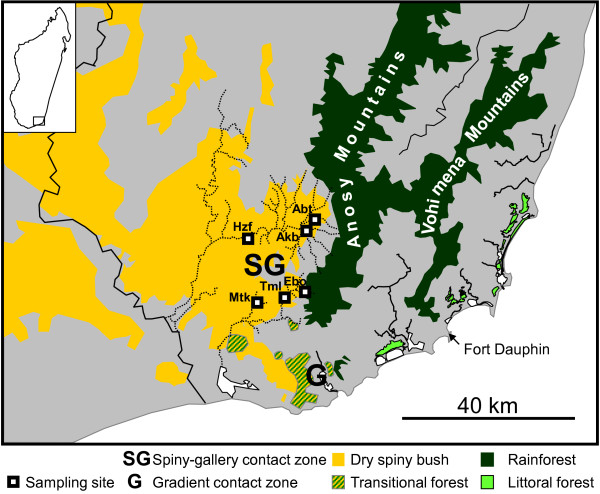
**Study area**. The figure displays the location of the study area in southeastern Madagascar, the locations of the two contact zones and a simplified schematic drawing of the distributions of major forest types. Actual forest cover is smaller due to fragmentation. Names of sampling sites are abbreviated as in Table 1.

**Figure 2:**
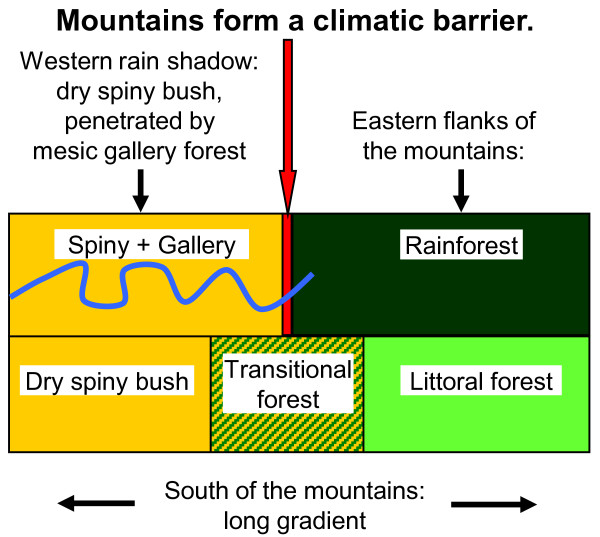
**Schematic view of the ecological settings in southeastern Madagascar**.

Gligor et al. [30] detected a hybrid zone between two species of mouse lemurs, *Microcebus griseorufus *and *M. murinus*, within a dry-humid ecotone in southeastern Madagascar. *Microcebus griseorufus *is a microendemic in the southern dry spiny bush [21,30-32]. *Microcebus murinus *has a very large distribution that comprises the western seasonally dry deciduous forest, southern gallery forest and southeastern humid littoral forest [21,30,33]. Southeastern Madagascar harbors a steep ecological gradient from the southern dry spiny bush to eastern humid forest (Figures 1 and 2). The north-southward directed Anosy- and Vohimena mountain chains form a climatic barrier to incoming clouds from the east. On their eastern flanks, there is rainforest. The vegetation shifts abruptly towards dry spiny bush in their western rain shadow. South of the southern tips of the mountain barrier, there is a wide ecological gradient from dry spiny bush in the west over intermediate transitional forest to humid littoral forest in the eastern part of the region. Gligor et al. [30] investigated the two species along a transect from dry spiny bush across the transitional forest into the littoral forest with the aid of nuclear microsatellites and sequences of a mitochondrial locus. They observed *Microcebus griseorufus *in the dry spiny bush and *M. murinus *in the humid littoral forest. In the transitional forest, they identified hybrids with *murinus-*like mitochondrial haplotypes and contrasting nuclear genotypes. Gligor et al. [30] concluded on an asymmetric introgression of *griseorufus-*like nuclear alleles into formerly autochthonous populations of *Microcebus murinus *in this intermediate vegetation zone between dry and humid vegetation.

Potential contact zones between species adapted to dry and mesic or humid conditions are not limited to such wide ecotonal gradients in Madagascar. For example, gallery forests with mesic conditions exist throughout the southern dry spiny bush, thus providing a virtually endless line of contact between different kinds of habitat.

It is unclear, which role hybridization could have played in Madagascar's diversification history. One key question towards a better understanding of its possible role is how far hybridization is influenced by ecological conditions. The aim of our study presented here was to contribute to the answering of the latter question by comparing contact zones of the same model species in different ecological settings. We questioned how far hybridization between *Microcebus griseorufus *and *M. murinus *would be restricted to the presence of an intermediate habitat where hybrids might have selective advantages. Would the same species also hybridize where they come into contact under different ecological conditions and would such a hybrid zone have a different structure? In order to answer these questions, we performed a population genetic study of the two species within the western rain shadow of the Anosy Mountains where spiny bush and gallery forest border each other sharply without intermediate habitats between them. We refer to this system as the spiny-gallery contact zone and to the hybrid zone in transitional forest as the gradient contact zone in the following (Figures 1 and 2).

Our genetic data yield evidence for hybridization in the spiny-gallery contact zone. Hybridization patterns in the two zones differ in several aspects. We discuss these differences and possible environment-related processes that could influence hybridization.

## Methods

### Description of the study area

The study area (Figure 1) is situated in and around parcel 2 of Andohahela National Park 20-40 km north of the hybrid zone in transitional forest investigated by Gligor et al. [30]. The area is dominated by dry spiny bush, which is crossed by temporary watercourses that mostly originate in the eastern humid forest. Along the watercourses, there are discontinuous narrow bands of gallery forests. Dry spiny bush and the more humid gallery forest border each other sharply without intermediate formations between them. We sampled mouse lemurs at six sites (Figure 1, Table 1). Rakotondranary et al. [34] performed a detailed ecological study of the mouse lemurs at these localities. At Hazofotsy (Hzf), we trapped all mouse lemurs in dry spiny bush. The closest remnants of gallery forest were in a distance of 1500 m. At Ambatoabo (Abt), we trapped mouse lemurs in a dense gallery forest and in adjacent dry spiny bush. At Ankoba (Akb), we trapped mouse lemurs in spiny bush, close to a gallery forest along the Azoara creek and near a small affluent. At Mangatsiaka (Mtk), there was spiny bush with sparse gallery vegetation along some temporary watercourses. In contrast to all other sites, these watercourses originate in the spiny bush and are dry most time of the year. We trapped mouse lemurs within the gallery and the surrounding spiny bush. At Tsimelahy (Tml), we trapped mouse lemurs in gallery forest along the Tarantsy River and a small affluent and in adjacent dry spiny bush. At Ebosika (Ebo), the Tarantsy river flows out of the eastern humid forest and crosses adjacent spiny bush with a gallery forest along the river. We captured mouse lemurs in spiny bush near the river and within the marginal part of the humid forest.

**Table 1:** Sampling

Site	Abbreviation	Habitat	Latitude	Longitude	Mg	Mm	Total
Hazofotsy	Hzf	S	-24.8356	46.5377	16	0	16
Ambatoabo	Abt	S,G	-24.8190	46.6696	0	19	19
Ankoba	Akb	S	-24.7958	46.6896	0	5	5
Mangatsiaka	Mtk	S,G	-24.9660	46.5574	12	63	75
Tsimelahy	Tml	S,G	-24.9556	46.6193	17	19	36
Ebosika	Ebo	S,H	-24.9439	46.6664	1	7	8
Total					46	113	159

S: dry spiny bush, G: gallery forest, H: humid forest, Mg and Mm: individuals with mitochondrial haplotypes of *Microcebus griseorufus *and *M. murinus *as revealed by phylogenetic reconstructions.

### Trapping of mouse lemurs and sample collection

We trapped mouse lemurs with banana-baited Sherman traps set up approximately 1 m above ground level. After anesthetizing the animals, we took small tissue samples from the ear for genetic analyses. We stored the samples in 90% ethanol at ambient temperature. We released all animals in the late afternoon of the same day at the respective sites of capture. We performed the trapping of mouse lemurs and the collection of tissue samples in compliance with respective research authorizations by the Malagasy Ministère de l'Environnement, des Eaux et Forêts (No 179/06/MINENV.EF/SG/DGEF/DPB/SCBLF/RECH, No 0174/07 -MINENV.EF/SG/DGEEF/DVRN/SPE) and by the Malagasy Ministère de l'Environnement, des Eaux et Forêts et du Tourisme (No 091/08/MEEFT/SG/DGEF/DSAP/SSE).

### Laboratory work

We isolated DNA from tissue samples using the DNeasy Blood & Tissue Kit (Qiagen) following the protocol for the purification of total DNA from animal tissues. We genotyped all individuals using the same genetic markers as Gligor et al. [30], which comprised the mitochondrial *hypervariable region 1 *(*HV1*) and nine nuclear microsatellite loci.

We amplified the *HV1 *with one primer binding in the constant region of the mitochondrial *D-loop *(mih1coau: 5'-GTTATAGTTTCAGGTTAGTCA-3') and one of the following primers binding in the *cytochrome b gene*: mih1cbau (5'-GATCTACTTATCCTTACATGA-3'), Mcytbf (5'-CTAGTAGAATGRATCTGAGG-3'), MrFTDcytbinfw58 (5'-GATTCTTCGCATTCCACTTC-3') or TsimMgCytbfw2 (5'-TCGGACAAGTGGCCTCTAT-3'). Typical PCR conditions comprised an initial denaturation step of 2 min at 92°C, 35 to 40 cycles of 40 s denaturation at 92°C, 60 s annealing at 55°C and 60 s elongation at 72°C, and one final elongation step of 5 min at 72°C. When using primer mih1cbau, we changed the annealing temperature to 54°C. When using primers Mcytbf or MrFTDcytbinfw58, we changed the elongation time to 70 s. We performed wax-mediated hot-start-PCR using the Qiagen Core Kit. For sequencing of the *HV1 *on both strands, we used the BigDye version 3.1 kit (Applied Biosystems) on a 3130 Genetic Analyzer (Applied Biosystems). We used mih1coau combined with either mih1cbau, TsimMgCytbfw2 or mih1cbin2Mz (5'-TTATACCWACYGTAAGYCTT-3') as primers for sequencing.

We applied the microsatellite markers *33104*, *Mm21*, *Mm22*, *Mm39*, *Mm51*, *Mm30*, *Mm42*, *Mm43b *and *Mm60 *[35] with the following modifications: We performed a first wax-mediated hot-start PCR with non-labeled primers and then reamplified 1-4 μl of the resulting product in repeated unidirectional extensions of one fluorescently labeled primer both with the Qiagen Core Kit. We changed the annealing temperature for locus *Mm42 *to 54°C. We used a 3130 Genetic Analyzer (Applied Biosystems) for electrophoresis and the GENEMAPPER 3.0 software (Applied Biosystems) for raw data analysis. We used 6-FAM and HEX as fluorescent dyes and GeneScan™-350 ROX™ (Applied Biosystems) as length standard.

### Data analysis: sequence data of the *HV1*

We aligned the sequences of the *HV1 *using the CLUSTAL W module implemented in BIOEDIT version 7.0.1 [36] and corrected the resulting alignment visually. We collapsed identical sequences to haplotypes using FABOX [37]. We performed phylogenetic tree reconstructions in order to assign haplotypes to the two species of mouse lemurs. To this aim, we added reference sequences of *Microcebus griseorufus *([GenBank: EU109652], [30]) and *M. murinus *([GenBank: DQ865143], [38]) to the dataset. For tree reconstructions based on maximum parsimony and Bayesian inference, we used a sequence of *Microcebus ravelobensis *([GenBank: AF285455], [39]) as outgroup.

We used PAUP* version 4.0b10 [40] for a maximum parsimony tree reconstruction. We performed a heuristic search with 100 random addition replicates and TBR branch swapping and a bootstrap analysis with 100 replicates for the evaluation of relative levels of support for internal nodes.

For the following tree reconstructions, we selected most appropriate substitution models for our data based on the Akaike Information Criterion as implemented in JMODELTEST version 0.1.1 [41,42]. We performed likelihood calculations for 24 models, which included 3 substitution schemes, equal or unequal base frequencies, a proportion of invariable sites and rate variation among sites with 4 rate categories on maximum likelihood optimized trees.

We used the MRBAYES software version 3.1.2 [43,44] for a tree reconstruction via Bayesian inference. We applied the HKY+G model in 2 independent analyses with 4 Markov Chain Monte Carlo chains and sampled the resulting trees every 100th generation. We stopped the analysis after 2,000,000 generations, when the standard deviation of split frequencies was 0.006532. We discarded the first 5,000 samples as burnin and summed the parameters from the remaining 15,000 samples. There was no increasing or decreasing trend in the log probabilities over generations. The potential scale reduction factor was 1.000 for all parameters. Based on these observations, we concluded that the analysis had converged. We discarded the first 5,000 trees as burnin and computed a consensus tree and posterior probabilities for internal nodes from the remaining 15,000 trees.

We used PHYML version 3.0 [42] for a maximum likelihood tree reconstruction, where we did not include an outgroup. For this dataset, JMODELTEST selected the HKY+G substitution model with a transition-transversion-parameter kappa of 80.0366 and a gamma shape parameter alpha of 0.0740. We applied the HKY+G model with 4 rate categories and fixed kappa and alpha to the values estimated by JMODELTEST. We performed tree searches using the SPR method with 5 random starting trees and the simultaneous NNI method with a BioNJ starting tree and selected the best tree overall with the aid of the BEST method. We evaluated support of internal branches by a bootstrap analysis with 100 replicates.

### Spatial overlap of individuals with different haplotypes

We compared the degree of spatial overlap between individuals with mitochondrial haplotypes of *Microcebus griseorufus *and *M. murinus *at Mangatsiaka and Tsimelahy as follows. For each individual with a *griseorufus-*like haplotype, we determined the 6 nearest neighbors based on pairwise distances between individual trapping positions rounded to the nearest 10 m (Additional file 1: Individual trap positions at Mangatsiaka and Tsimelahy). We then calculated the average proportion of *murinus-*like haplotypes in the nearest neighborhood of *griseorufus-*like individuals for each sampling site. In order to evaluate a potential impact of unequal numbers of individuals with the two species' haplotypes, we calculated averages from 100 datasets, where we reduced the greater group to the same size as the smaller group by random resampling. We wrote the program SOA [45] for these analyses.

We further tested for significant differences of the proportions of *murinus-*like and *griseorufus-*like mitochondrial haplotypes among individuals captured at Mangatsiaka and Tsimelahy with the aid of Fisher's exact test as implemented in PASW Statistics 17.0.

### Microsatellite data: F-statistics, tests of deviations from Hardy-Weinberg equilibrium and linkage disequilibria

For the following tests, we divided the individuals into local samples according to sampling sites and *griseorufus-*like (-Mg) or *murinus-*like (-Mm) mitochondrial haplotypes. We excluded some resulting small samples with sample sizes between 1 and 7 and included the following ones: Hzf-Mg (n = 16), Abt-Mm (n = 19), Mtk-Mg (n = 12), Mtk-Mm (n = 63), Tml-Mg (n = 17) and Tml-Mm (n = 19). We used FSTAT version 2.9.3.2 [46] for the following analyses. We estimated global F-statistics and F_ST _between pairs of samples according to Weir and Cockerham [47]. We tested for deviations from Hardy-Weinberg equilibrium for each locus and over all loci in each sample with 54,000 permutations of the original data. We tested for linkage disequilibria for all pairs of loci in each sample with the aid of LINKDOS [48] as implemented in GENETIX version 4.05.2 [49].

### Identification of hybrids

We used two Bayesian methods implemented in the programs STRUCTURE version 2.1 [50] and NEWHYBRIDS version 1.1 [51] to identify hybrids based on individual microsatellite genotypes in comparison to mitochondrial haplotypes. We used three datasets of microsatellite genotypes: One large dataset comprised all individuals in the study; two smaller ones comprised all individuals at Mangatsiaka and all individuals at Tsimelahy.

With NEWHYBRIDS, we estimated posterior probabilities to belong to one of six predefined categories of purebreds and hybrids for each individual's genotype. We used the default genotype frequency class file with the following categories: Mg (purebred *Microcebus griseorufus*), Mm (purebred *M. murinus*), F1 (Mg × Mm), F2 (F1 × F1) and two classes of first generation backcrosses Mg-Bx1 (F1 × Mg) and Mm-Bx1 (F1 × Mm). We performed Markov chain Monte Carlo computations with a burnin period of 100,000 steps and a post-burnin period of 1,000,000 steps with Jeffreys-like priors for the mixing proportions and allele frequencies and without prior population or allele frequency information. As recommended by Vähä and Primmer [52], we used a threshold of 0.5 as criterion for the detection of hybrids and the distinction between hybrid categories. We regarded an individual as purebred when the posterior probability to be purebred from the species corresponding to its mitochondrial haplotype was > 0.5, as a hybrid when it was ≤0.5 and as a specific category of hybrid when the corresponding posterior probability was > 0.5.

STRUCTURE performs a Bayesian clustering of genotypes into a number of clusters K predefined by the user and estimates membership coefficients as posterior probabilities for each genotype to belong to each of the K clusters. We used the admixture ancestry model with independent allele frequencies and without prior population information. We fixed the allele frequency parameter lambda to one and let the program estimate a uniform value for the model parameter alpha. We performed 10 independent runs with a burnin of 10,000 generations and a post-burnin period of 40,000 generations for different values of K from 1 to 9 for the large dataset and from 1 to 5 for the two smaller datasets. We then used the ad hoc statistic ΔK [53] to determine the most appropriate number of clusters for our data. ΔK unambiguously indicated K = 2 as most appropriate for all datasets. We then performed optimal alignments of the results from the 10 independent runs with K = 2 for each dataset and calculated average membership coefficients using the full-search algorithm of CLUMPP version 1.1.1 [54]. We applied a threshold of 0.1 to the membership-coefficients as criterion for hybrid detection. We regarded an individual as purebred when the average membership coefficient for the cluster corresponding to its mitochondrial haplotype was > 0.9 and as a hybrid when it was ≤ 0.9.

### Analyses of simulated microsatellite data

In order to evaluate the power of our microsatellite data to detect hybrids and to distinguish between different hybrid classes, we analyzed simulated datasets with STRUCTURE and NEWHYBRIDS. Within our original microsatellite genotypes, we identified all individuals observed as purebred from the species corresponding to their mitochondrial haplotypes with probabilities > = 0.9 both with STRUCTURE and NEWHYBRIDS. We then used these individuals' genotypes to create simulated datasets with the software HYBRIDLAB version 1.0 [55]. We analyzed three sets of simulated data:

Simulation A: We simulated 100 datasets, which each contained genotypes of 100 Mg (purebred *Microcebus griseorufus*), 100 Mm (purebred *M. murinus*), 10 F1 (Mg × Mm) and 10 F2 (F1 × F1). Each file further contained genotypes that we call first generation backcrosses, 10 Mg-Bx1 (F1 × Mg) and 10 Mm-Bx1 (F1 × Mm) and second generation backcrosses, 10 Mg-Bx2 (Mg-Bx1 × Mg) and 10 Mm-Bx2 (Mm-Bx1 × Mm). We analyzed all simulated datasets with STRUCTURE and NEWHYBRIDS under the same settings as our real data. With STRUCTURE, we performed 10 independent runs with K = 2 for each dataset and summed up the results with CLUMPP. We evaluated different threshold values of 0.3, 0.2 and 0.1 for hybrid detection. With NEWHYBRIDS, we used a threshold value of 0.5 as with the real data. We then calculated efficiency and accuracy similar to Vähä and Primmer [52]. Efficiency is the proportion of individuals of a certain category correctly identified (e.g. true F1-hybrids identified as hybrids), and accuracy is the proportion of individuals assigned to a certain category that was correctly identified (e.g. individuals identified as purebred *M. griseorufus *that are truly purebred of that species).

Simulation B: At Mangatsiaka, we had sampled 12 individuals with *griseorufus-*like and 63 with *murinus-*like mitochondrial haplotypes. We questioned if the number of hybrids that we observed in the real data at Mangatsiaka could be an artifact due to these unequal sample sizes. We simulated a scenario where all individuals at Mangatsiaka are purebred with the aid of 100 datasets, which comprised each 12 purebred *Microcebus griseorufus *and 63 purebred *M. murinus*. We analyzed these datasets under the same settings as the real data with both programs and calculated the proportions of datasets where the number of false-positive hybrids was as great as or greater than the number of hybrids detected in the real data.

Simulation C: In the real data, we had identified several individuals as hybrids with only one of both programs or in only one of two datasets. We questioned if such discrepancies more probably indicated false-positive or false-negative hybrids. We used real genotypes of purebred *Microcebus griseorufus *and *M. murinus *from Tsimelahy to simulate 100 local datasets with the same size and composition as in simulation A. We then enlarged each dataset by adding 100 purebred individuals of each species, which we simulated based on purebred genotypes from Hazofotsy and Ambatoabo. We analyzed all local and enlarged datasets with both programs under the same settings as in simulations A and B and evaluated the proportions of true hybrids among classifications that were discrepant between programs or datasets. We then combined the evidence from both programs and corresponding datasets by accepting all individuals as hybrids that we identified as such at least once and calculated efficiencies and accuracies.

## Results

### Mitochondrial haplotypes and phylogenetic reconstructions

**Figure 3:**
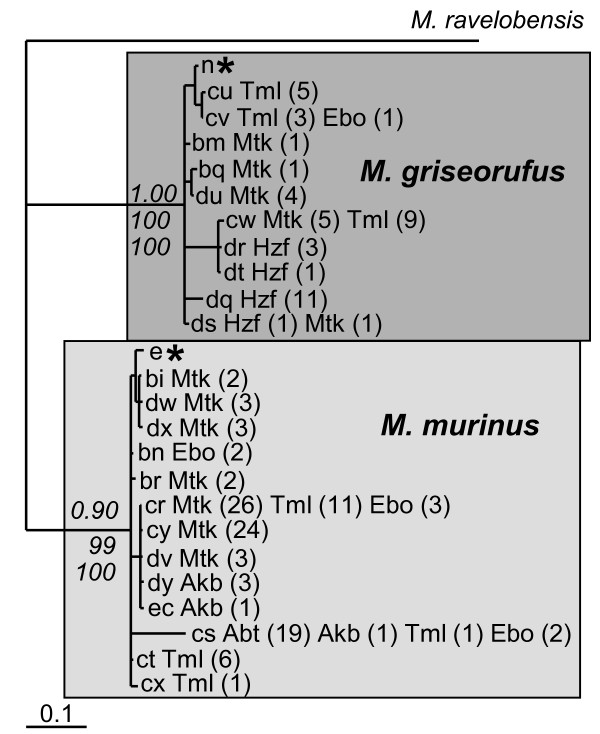
**Tree reconstruction based on *HV1 *sequences**. The figure displays a tree reconstruction via Bayesian inference. Haplotype identifiers: lowercase characters followed by the sampling site abbreviation as in Table 1 and the number of individuals in brackets. Asterisks denote reference haplotypes for the two species of mouse lemurs. Numbers in italics are support values for the two major clades in the topology based on Bayesian inference and bootstrap values from the maximum parsimony and maximum likelihood tree reconstructions. Support values for internal nodes in the two major clades are not shown.

We observed 23 different haplotypes among the 159 individuals [GenBank:JF510161-JF510319] (See also Additional file 2: Genotypes.). Figure 3 displays a tree reconstruction based on Bayesian inference. All tree reconstructions yielded two well-supported major clades with Bayesian posterior probabilities of 1.00 and 0.90, maximum parsimony bootstrap values of 100 and 99 and maximum likelihood bootstrap values of 100 each. The clades had identical haplotype compositions in all tree reconstructions and each one contained one of the two reference haplotypes. We could thus unambiguously assign all haplotypes to either *Microcebus griseorufus *or *M. murinus*. We assigned 10 haplotypes, which represented 46 individuals, to *Microcebus griseorufus *and 13 haplotypes, which represented 113 individuals, to *M. murinus*.

### Spatial distribution of mitochondrial haplotypes

**Figure 4:**
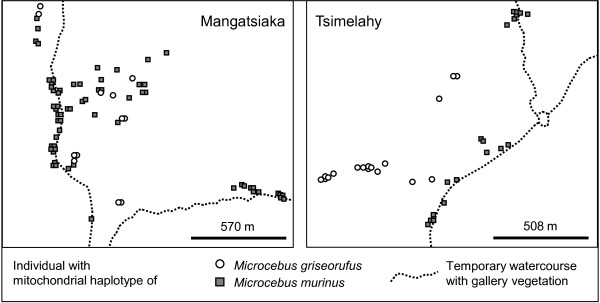
**Distributions of individuals with haplotypes of different species at Mangatsiaka and Tsimelahy**.

The spatial distribution of haplotypes of the two species displayed a pattern of close association with different habitats. Haplotypes of *Microcebus griseorufus *occurred in spiny bush exclusively 760 to 1310 m from the nearest watercourse at Hazofotsy, 70-460 m at Mangatsiaka, 110-650 m at Tsimelahy and 250 m at Ebosika. Haplotypes of *Microcebus murinus *occurred at all sites with gallery forest and were absent at Hazofotsy, where we set traps only in spiny bush (Table 1, Figures 1 and 3). We observed the majority of individuals with *murinus-*like haplotypes in gallery forest. They occurred also in the adjacent spiny bush with distances to the nearest watercourse up to 260 m at Ambatoabo, 90 m at Ankoba, 680 m at Mangatsiaka, 130 m at Tsimelahy and 460 m at Ebosika. At Ebosika, we found some individuals in marginal parts of adjacent rainforest up to 150 m from the margin. We observed haplotypes of both species at Mangatsiaka, Tsimelahy and Ebosika. There was considerable spatial overlap of both species within the spiny bush at Mangatsiaka, but not at Tsimelahy (Figure 4). We could not further investigate this at Ebosika, where only one individual with a *griseorufus-*like haplotype occurred. With some caution, we can use the mitochondrial data as indirect clues on differences of relative abundances between sites. The proportions of individuals with *murinus- *and *griseorufus-*like haplotypes at Mangatsiaka (Mtk) and Tsimelahy (Tml) were significantly different according to Fisher's exact test when including all individuals (Mtk_all_:63/12, Tml_all_:19/17, two-sided p_all_: 0.001) and when including only individuals captured more than 50 m from the nearest watercourse (Mtk_spiny_: 26/12, Tml_spiny_: 3/17 Mg, two-sided p_spiny_: 0.000). The same held true after exclusion of individuals identified as hybrids (Mtk_all_: 54/8, Tml_all_: 19/16 two-sided p_all_: 0.001 and Mtk_spiny_: 24/8, Tml_spiny_: 3/16 two-sided p_spiny_: 0.000). These observations suggest that *Microcebus murinus *predominates at Mangatsiaka while both species appear to be similarly abundant at Tsimelahy. In the spiny bush more than 50 m from the nearest watercourse, relative abundances seem to have inverse proportions with a majority of *Microcebus murinus *at Mangatsiaka and of *M. griseorufus *at Tsimelahy. The average proportion of *murinus-*like haplotypes among the 6 nearest neighbors of *griseorufus-*like individuals was 68% at Mangatsiaka and 20% at Tsimelahy. The average diameters of neighborhoods were very similar at both sites with 259 m at Mangatsiaka and 245 m at Tsimelahy. Resampling to equal sample sizes with both species' haplotypes reduced the proportion of *murinus-*like haplotypes in the neighborhood of *Microcebus griseorufus *to 46% at Mangatsiaka and 19% at Tsimelahy. Elimination of all *murinus-*like individuals trapped more than 50 m from the nearest watercourse at Mangatsiaka reduced it to 50% and additional resampling to equal numbers of both species' haplotypes to 42%. We observed similar differences between these sites with different numbers of nearest neighbors included in the calculations (Additional file 3: Proportion of *Microcebus murinus *within the nearest neighborhood of *M. griseorufus *and Additional file 4: Diameters of neighborhoods with different numbers of nearest neighbors). In summary, these results indicate that there is considerably more spatial overlap between individuals with *murinus- *and *griseorufus-*like haplotypes at Mangatsiaka than at Tsimelahy. The greater overlap at Mangatsiaka is influenced both by the greater relative abundance of *murinus-*like individuals and by their stronger invasion of the spiny bush.

### Deviations from Hardy-Weinberg equilibrium and linkage disequilibria

Global F-statistics indicated heterozygote deficiency on the global level (F_IT _= 0.192), which was more due to heterozygote deficiency within local samples (F_IS _= 0.137) than to differences of allele frequencies between samples (F_ST _= 0.063). Pairwise F_ST _values were small (0.0055-0.0339) between samples with mitochondrial haplotypes of the same species and considerably greater (0.0861-0.1128) between samples with haplotypes of different species (Table 2). We observed significant heterozygote deficiencies in tests over all loci for individuals with *griseorufus- *and *murinus-*like mitochondrial haplotypes at Mangatsiaka and for individuals with *murinus*-like haplotypes at Ambatoabo and Tsimelahy with F_IS _values in a range from 0.101 to 0.210 (Table 3). Tests for single loci were more often significant in samples with *murinus-*like haplotypes than in those with *griseorufus-*like haplotypes (Table 3). We observed several pairs of loci in significant linkage disequilibrium after strict Bonferroni correction in each sample (Table 3). Individuals with *murinus-*like haplotypes at Mangatsiaka displayed the greatest number of significant locus pairs (16 of 36). Although significant, linkage disequilibria were not strong in most cases: The respective coefficients of correlation were below 0.4 in 95% of the significant tests.

**Table 2:** Pairwise F_ST _between samples

	Hzf-Mg	Mtk-Mg	Tml-Mg	Abt-Mm	Mtk-Mm
Mtk-Mg	0.0078				
Tml-Mg	0.0339	0.0161			
Abt-Mm	0.1045	0.0953	0.1001		
Mtk-Mm	0.1030	0.0861	0.0907	0.0170	
Tml-Mm	0.1128	0.0946	0.1013	0.0104	0.0055

Hzf, Mtk, Tml, Abt: abbreviations of sampling sites; Mg: individuals with *griseorufus-*like mitochondrial haplotypes, Mm: individuals with *murinus*-like mitochondrial haplotypes.

**Table 3:** Tests of heterozygote deficiency and linkage disequilibria

	Hzf-Mg	Abt-Mm	Mtk-Mg	Mtk-Mm	Tml-Mg	Tml-Mm
F_IS _33104	0.085	-0.069	-0.115	0.023	0.013	-0.003
F_IS _Mm21	0.000	-0.050	0.438*	0.110	0.041	-0.029
F_IS _Mm22	-0.047	0.455*	-0.034	0.290*	0.024	0.158
F_IS _Mm30	0.122	0.320	0.436	0.228*	-0.120	0.269
F_IS _Mm39	-0.157	0.080	-0.071	0.009	0.109	0.233
F_IS _Mm42	0.045	0.356	0.369	0.327*	0.032	0.216
F_IS _Mm43	0.109	0.632*	0.513	0.316*	0.563*	0.302
F_IS _Mm51	0.068	0.348	-0.229	0.126	0.204	-0.304
F_IS _Mm60	0.007	-0.061	-0.017	0.075	-0.106	-0.038
F_IS _All Loci	0.023	0.210*	0.139*	0.166*	0.083	0.101*
LD	9	12	11	16	5	7

The table displays F_IS _for each locus, F_IS _over all loci and the number of locus pairs in significant linkage disequilibrium for each sample. Hzf, Abt, Mtk, Tml: abbreviations of sampling sites; Mg: individuals with *griseorufus-*like mitochondrial haplotypes, Mm: individuals with *murinus*-like mitochondrial haplotypes. Asterisks denote significant heterozygote deficiency after strict Bonferroni correction. Nominal level: 0.05, adjusted p for tests for each locus in each sample: 0.00093, adjusted p for tests over all loci: 0.00833. P-values were obtained after 54,000 randomizations of the original data. No significant heterozygote excess was observed. LD: number of locus pairs in significant linkage disequilibrium after strict Bonferroni correction (nominal level: 0.05, adjusted p: 0.00023).

### Identification of hybrids

**Figure 5:**
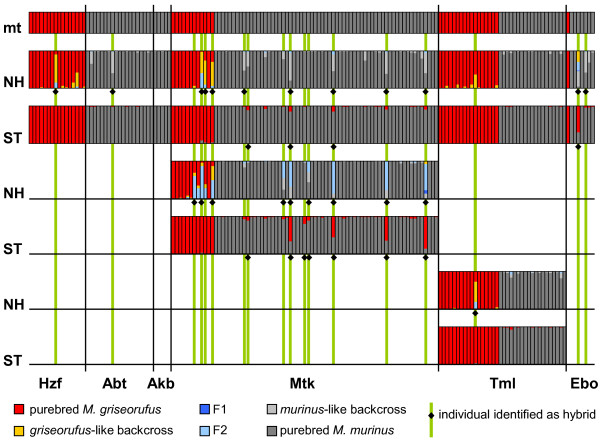
**Analyses of three datasets of microsatellite genotypes with STRUCTURE and NEWHYBRIDS**. Datasets: all individuals, individuals at Mangatsiaka (Mtk) and individuals at Tsimelahy (Tml). Each vertical bar represents one individual. Different colors represent posterior probabilities to be member of a group based on microsatellite genotypes. mt: mitochondrial haplotypes. Colors represent the two species. NH: NEWHYBRIDS, ST: STRUCTURE, Hzf, Abt, Akb, Mtk, Tml, Ebo: abbreviations of sampling sites as in Table 1.

We identified 18 of the 159 individuals as hybrids in one or several analyses (Figure 5). Although there was a high level of congruence between the different analyses, the individual identification of hybrids differed in cases between datasets and programs. Within the great dataset, we identified 13 individuals as hybrids. Within the two local datasets of Mangatsiaka and Tsimelahy, we identified 5 additional individuals. Twelve identified hybrids had mitochondrial haplotypes of *Microcebus murinus*, 6 of *M. griseorufus*. All hybrids identified by STRUCTURE had mitochondrial haplotypes of *Microcebus murinus*. NEWHYBRIDS identified hybrids with both species' haplotypes. Hybrids with *griseorufus-*like haplotypes were restricted to the spiny bush, hybrids with *murinus-*like haplotypes occurred in the spiny bush and in gallery forest. Hybrids were unevenly distributed over sampling sites: 13 of 18 hybrid individuals were observed at Mangatsiaka. Backcross- and F2-signals dominated among the hybrid classes indicated by NEWHYBRIDS. No individual was assigned to the class of F1 hybrids.

### Analyses of simulated microsatellite data

Both, STRUCTURE and NEWHYBRIDS identified hybrids with great accuracy and purebreds with slightly smaller accuracy in simulation A (Tables 4 and 5). Efficiency was great for purebreds but considerably smaller for hybrids, particularly first- and second generation backcrosses (Mg-Bx1, Mm-Bx1, Mg-Bx2, Mm-Bx2), which were often misclassified as purebreds (Tables 4, 5 Additional file 5: Simulation A, STRUCTURE, observed proportions with a threshold of 0.1). The efficiency of hybrid detection with STRUCTURE decreased dramatically when we used threshold values greater than 0.1, whereas accuracies did not increase considerably (Table 4). We therefore concluded that the threshold value of 0.1, which we had applied to our real data, was an optimal choice. Beyond the detection of hybrids as such, the distinction between specific hybrid categories with NEWHYBRIDS was less efficient and less accurate (Table 5). We can thus not determine with certainty, which category the hybrids identified in our real data truly belong to. This would presumably require a greater number of loci [52]. At least we can conclude that F1-hybrids are probably rare because there are almost no signals for this class in the real data, whereas simulation A indicates that we should correctly identify more than half of them.

**Table 4:** Simulation A, STRUCTURE, efficiency and accuracy with different threshold values

Threshold	0.1	0.2	0.3
Efficiency Mg	**1.000**	1.000	1.000
Efficiency Mm	**0.998**	1.000	1.000
Efficiency F1	**0.980**	0.912	0.811
Efficiency F2	**0.923**	0.791	0.614
Efficiency Mg-Bx1	**0.537**	0.325	0.177
Efficiency Mm-Bx1	**0.524**	0.329	0.206
Efficiency Mg-Bx2	**0.174**	0.062	0.026
Efficiency Mm-Bx2	**0.185**	0.061	0.022
Accuracy Mg	**0.881**	0.849	0.825
Accuracy Mm	**0.882**	0.852	0.832
Accuracy Hyb	**0.994**	1.000	0.999

The table displays efficiencies and accuracies for three different thresholds for the posterior probabilities used to distinguish between purebreds and hybrids. Efficiency: efficiency of identifying purebreds as purebreds and hybrids of different categories as hybrids, accuracy: accuracy of identified purebreds and hybrids, Mg: purebred *Microcebus griseorufus*, Mm: purebred *M. murinus*, F1: Mg × Mm, F2: F1 × F1, Mg-Bx1: F1 × Mg, Mm-Bx1: F1 × Mm, Mg-Bx2: Mg-Bx1 × Mg, Mm-Bx2: Mm-Bx1 × Mm, Hyb: hybrid, bold: values for the threshold of 0.1, which we applied to the real data.

**Table 5:** Simulation A, NEWHYBRIDS, efficiency and accuracy

	Mg	Mm	Hybrid	F1	F2	Mg-Bx1	Mm-Bx1	**H.u.c**.
Mg (n = 10,000)	**0.998**	0.000	0.002	0.000	0.000	0.002	0.000	0.000
Mm (n = 10,000)	0.000	**0.986**	0.014	0.000	0.000	0.000	0.012	0.002
F1 (n = 1000)	0.000	0.003	**0.997**	**0.632**	0.010	0.091	0.053	0.211
F2 (n = 1000)	0.006	0.012	**0.982**	0.075	**0.346**	0.233	0.218	0.110
Mg-Bx1 (n = 1000)	0.164	0.002	**0.834**	0.064	0.030	**0.655**	0.002	0.083
Mm-Bx1 (n = 1000)	0.000	0.208	**0.792**	0.049	0.048	0.004	**0.629**	0.062
Mg-Bx2 (n = 1000)	0.510	0.000	**0.490**	0.004	0.006	**0.454**	0.000	0.026
Mm-Bx2 (n = 1000)	0.000	0.542	**0.458**	0.009	0.011	0.000	**0.413**	0.025
Accuracy	0.936	0.928	0.966	0.759	0.767	0.763	0.729	

The table displays the proportions of simulated individuals assigned to different categories, efficiencies and accuracies. Columns: observed categories, rows: true categories, last row: accuracies, bold: efficiencies, Mg: purebred *Microcebus griseorufus*, Mm: purebred *M. murinus*, F1: Mg × Mm, F2: F1 × F1, Mg-Bx1: F1 × Mg, Mm-Bx1: F1 × Mm, Mg-Bx2: Mg-Bx1 × Mg, Mm-Bx2: Mm-Bx1 × Mm, H.u.c.: hybrid of unclear category. We did not set up additional observed categories for second generation backcrosses in the genotype frequency class file used with NEWHYBRIDS. For this reason, we counted Mg-Bx2 assigned to Mg-Bx1 and Mm-Bx2 assigned to Mm-Bx1 as correctly identified.

The simulated scenario of no hybridization at Mangatsiaka appears as highly improbable with respect to the results of simulation B (Additional file 6: Simulation B: Scenario of no hybrids at Mangatsiaka). Most simulated purebred individuals were correctly identified with both programs. The number of purebred *Microcebus murinus *misclassified as hybrids was smaller than the number of hybrids with *murinus-*like haplotypes detected in the real data with both programs in all simulated datasets. The respective number for *Microcebus griseorufus *was reached in only 4% of the simulated datasets with NEWHYBRIDS.

Almost all discrepancies between programs and datasets in simulation C concerned true hybrids that remained undetected with one of the programs or in one of two datasets (Table 6). Accepting all hybrids identified with at least one program in at least one dataset led to a considerable increase of hybrid detection efficiency while the hybrid accuracy remained very high. Even with this approach, several true hybrids remained completely undetected in every dataset, and we always underestimated the true number of hybrids. Based on these findings, we conclude that most of the 18 hybrids that we identified in the real data are probably true hybrids and that we probably underestimate their true number.

**Table 6:** Simulation C, discrepancies between datasets and programs

Combination	A	B	C	D	E
Program	NEW- HYBRIDS	STRUCTURE	Both programs	Both programs	Both programs
Datasets	L+E	L+E	L	E	L+E
Efficiency Mg	1.000	1.000	1.000	1.000	1.000
Efficiency Mm	0.999	1.000	0.999	1.000	0.999
Efficiency F1	1.000	1.000	1.000	1.000	1.000
Efficiency F2	0.990	0.960	0.988	0.976	0.990
Efficiency Mg-Bx1	0.883	0.688	0.868	0.852	0.883
Efficiency Mm-Bx1	0.884	0.696	0.877	0.820	0.884
Efficiency Mg-Bx2	0.547	0.279	0.504	0.502	0.547
Efficiency Mm-Bx2	0.532	0.248	0.514	0.454	0.532
Accuracy Hyb	0.999	0.999	0.999	1.000	0.998
Accuracy Mg	0.946	0.905	0.941	0.939	0.946
Accuracy Mm	0.944	0.902	0.942	0.931	0.944
Discrepant between sets	325	426			715
Disc. true Hyb	97.8%	99.5%			98.7%
Discrepant between programs			1098	949	1363
Disc. true Hyb			99.4%	99.8%	99.3%
N Hyb obs. per set/pair sets	41-57	30-46	40-55	37-55	41-57

We accepted all individuals as hybrids that were identified in at least one of two corresponding datasets (combinations A and B), by at least one program (combinations C and D) and by at least one program in at least one of two corresponding datasets (combination E). Datasets: L: local sets (n = 100), E: enlarged sets (n = 100), L+E: combined evidence from corresponding local and enlarged sets; Discrepant between sets: individuals identified as hybrids in only one of two corresponding datasets; Discrepant between programs: individuals identified as hybrids with only one of the two programs; Disc true Hyb: percentage of true hybrids among discrepant individuals in the line above; N Hyb obs. per set/pair sets: range of observed numbers of hybrids in 100 datasets or pairs of corresponding datasets. The true number of hybrids per set was 60. We disregarded the 20,000 additional purebred individuals comprised in the enlarged datasets exclusively for the calculation of efficiencies, accuracies and numbers of discrepant individuals. Only six of these individuals were misclassified as hybrids in combination C.

## Discussion

Our results demonstrate that *Microcebus griseorufus *and *M. murinus *hybridize within the spiny-gallery contact zone. In the following, we discuss our findings from this area and compare them to those from the gradient contact zone. We refer to Gligor et al. [30] for a more detailed characterization of the latter.

### Environmental settings

The environmental settings are very different in both contact zones. In the spiny-gallery zone, a network of narrow gallery forests penetrates the dry spiny bush. Both habitats border each other sharply without intermediate zones between them. In the gradient contact zone, there is a large climatic gradient with an approximately 10 km wide intermediate zone with transitional forests between dry spiny bush west of it and humid littoral forests to the east.

### Local admixture of mitochondrial haplotypes

According to the distributions of mitochondrial haplotypes in our study area, *Microcebus griseorufus *appears as strictly bound to the spiny bush and *M. murinus *as closely associated with the gallery forest. The fact that the latter also invades adjacent habitats of different types leads to local sympatric admixture within the spiny bush at Mangatsiaka. This is in contrast to observations at other places. At Beza Mahafaly in southwestern Madagascar, Heckman et al. [32] observed only *griseorufus-*like mitochondrial haplotypes despite morphological evidence for the presence of both species [21,56]. At Berenty, west of our study area, Yoder et al. [31] observed mitochondrial haplotypes of *Microcebus murinus *in gallery forests and of *M. griseorufus *in adjacent spiny bush without admixture. In the gradient contact zone investigated by Gligor et al. [30], *griseorufus-*like haplotypes where restricted to spiny bush and *murinus-*like haplotypes to transitional and littoral forest.

### Directionality of hybridization

Other than in the gradient contact zone, we observed hybrids with mitochondrial haplotypes of both species in the spiny-gallery contact zone. The number of detected hybrids indicates a limited degree of hybridization although we might have underestimated it. F1-hybrids are apparently rare. It appears thus as probable that F2-hybrids are rare as well and that most hybrids are backcrosses or crosses between backcrosses. This would indicate that the formation of F1-hybrids is more difficult than the interbreeding of hybrids with purebreds or with other hybrids, which would provide opportunities for introgression as soon as a few hybrids exist [2,57]. Our results point to a limited degree of bidirectional introgressive hybridization, which would also be in line with the observation of heterozygote deficiencies and linkage disequilibria in subsamples of both species separated according to their mitochondrial haplotypes. The directionality of introgressive hybridization is not to be confounded with the directionality of habitat-invasion. The latter is apparently unidirectional because *Microcebus murinus *invades the spiny bush, whereas we have no evidence for an invasion of the gallery forest by *M. griseorufus*.

Gligor et al. [30] did not apply a threshold value for hybrid detection when using STRUCTURE. In their study, ΔK [53] indicated K = 2 as the most probable uppermost structure but there was also some support for K = 3. We re-evaluated the original results of Gligor et al. [30] with K = 2 and applied a threshold of 0.1 as in our study presented here. Among 38 individuals from the transition zone, we identified 28 hybrids in a large dataset and 35 hybrids in a smaller dataset (Additional file 7: Re-evaluated identification of hybrids in the gradient contact zone). All individuals in the spiny bush and littoral forest were classified as purebred. With K = 3, most individuals from the transition zone formed a third cluster of genotypes, while those from the spiny bush and littoral forest were assigned to the other two clusters [30]. None of the *murinus-*like mitochondrial haplotypes in the transition zone occurred in the littoral forests further east. Based on this observation and their results from microsatellite data, Gligor et al. [30] concluded on a unidirectional introgression of *griseorufus-*like nuclear alleles into autochthonous populations of *Microcebus murinus *in the transition zone.

### Asymmetry of hybridization and mechanism of secondary contact

Despite bidirectionality, hybridization in the spiny-gallery contact zone is apparently asymmetric since the majority of hybrids carry *murinus*-like mitochondrial haplotypes, which is similar to the gradient contact zone, where all hybrids carry *murinus-*like haplotypes. In the spiny-gallery zone, secondary contact occurs most probably within the habitat of *Microcebus griseorufus*, which is invaded by *M. murinus*. In the gradient contact zone, the respective mechanism is unknown since Gligor et al. [30] did not detect recent purebred immigrants in either direction. They discussed cyto-nuclear incompatibility [58,59] versus environmental selection, potentially on cyto-nuclear gene complexes (e.g. [60]), as possible causes for asymmetric introgression. Our findings in the spiny-gallery contact zone now contradict strict incompatibility of either species' mitochondrial genome with a hybridized nuclear background. Locally unequal abundances of both species in the spiny-gallery contact zone could play a role for asymmetric hybridization. This is most obvious at Mangatsiaka, where we observed most hybrids and where *Microcebus murinus *appears to be more abundant than its congener.

*Microcebus griseorufus *appears as a specialist restricted to the southern spiny bush and *M. murinus *as a generalist that colonizes a wider range of habitats within an extremely large range from northern to southeastern Madagascar. In general, widespread species tend to be locally more abundant than species with restricted ranges. This has been explained by metapopulation dynamics [61] and by Brown's [62] niche-breadth hypothesis, which predicts that among closely related species, those with the broader niche will be both locally more abundant and more widespread. It remains, however, questionable if greater ecological plasticity alone can explain the apparent success of *Microcebus murinus *at Mangatsiaka. According to Ganzhorn and Schmid [63], the species is extremely sensitive to altered environmental conditions. They compared populations in primary and secondary dry deciduous forest in western Madagascar. In secondary forest, *Microcebus murinus *displayed lower population densities and extremely small year-to-year survival rates. The authors explained this by higher ambient temperatures and lower availability of tree holes, which decreased the possibilities for energy-saving daily torpor and hibernation.

### Evidence for environmental factors influencing hybridization

The significant linkage disequilibria and heterozygote deficiencies in the spiny-gallery zone could most likely point to selection against or in favor of certain allelic combinations in the course of introgressive hybridization. Theoretically, they could also be produced by recent intraspecific admixture, e.g. in the course of colonization-recolonization dynamics, but, with respect to very small pairwise F_ST _values between samples of the same mitochondrial species, such a scenario appears not very plausible. As well, in the gradient-contact zone, significant heterozygote deficiencies and linkage disequilibria, which were mostly restricted to the transition zone, pointed to selection [30]. Environmental selection appears as probable in both zones because genetic patterns are congruent with habitat patterns. The distribution of purebreds and hybrids in the spiny-gallery zone closely reflects the mosaic-like distribution of spiny bush and gallery forest. In the gradient zone, three clusters of genotypes observed with STRUCTURE with K = 3 were largely congruent with the three vegetation zones along the west-eastern climatic gradient [30]. With K = 2, membership coefficients were predominantly *griseorufus-*like in the western part of the transition zone and predominantly *murinus-*like in the eastern part (Additional file 7: Re-evaluated identification of hybrids in the gradient contact zone). Genetic distances between sites were significantly correlated with vegetation zone differences under control of geographic distances [30].

### Possible environment-related processes in the two contact zones

A remaining open question is why we did observe heterozygote deficiencies and linkage disequilibria at several sampling sites in the spiny-gallery zone, where we identified no or few hybrids. One could explain this by the difficult detection of hybrids, which might be more pronounced in smaller local samples. Another potential explanation could be temporal change of the frequency of hybridization, which could be due to stochastically fluctuating environmental conditions. Indeed, there is some evidence for strong environmental stochasticity that could have an important impact on hybridization in the spiny-gallery contact zone. According to Dewar and Richard [64], Madagascar has significantly less predictable rainfall than continental Africa. In the North and the South, this unpredictability takes the form of high interannual variation of total precipitation. The weather station at Behara, which is situated 18 km west of Mangatsiaka in our study area, displayed a mean annual rainfall of 532 mm and the least predictable climate among 15 weather stations throughout Madagascar included in the study of Dewar and Richard [64]. The climate in our study area is characterized by a short rainy season and a long dry season (e.g. [65]). The region is irregularly struck by severe droughts, which can last one or several subsequent years [66-68]. According to Elmqvist et al. [69], the frequency of droughts in southern Madagascar is increasing since the 1970's (but see [68]). Without long-term data from our study area, we can only speculate how these dramatic fluctuations of rainfall might influence hybridization. Neaves et al. [7] discuss density fluctuations of sympatric grey kangaroos due to fluctuations in rainfall as potential cause of occasional hybridization. A hypothetical scenario for our study area could be that density fluctuations due to irregular rainfall are most pronounced for *Microcebus murinus*, which is adapted to more mesic conditions. Such fluctuations could even follow different rhythms or have different amplitudes near gallery forests with headwaters in the spiny bush or in the rainforest. Occasional high densities would lead to increased overlap with *Microcebus griseorufus *and subsequent hybridization. Intermittent droughts could reduce the density of *Microcebus murinus*, reduce invasive pressure and shift the adaptive landscape towards more divergent selection, which could help to maintain species integrity.

Pollen data from southern Madagascar [70] and subfossil remains at Andrahomana cave [71-73] indicate that the vegetation zones in the gradient contact zone could have shifted in eastward direction in the course of long-term aridification during the last 3000 years approximately. Accordingly, Gligor et al. [30] propose that such a shift of the adaptive landscape would have provided a growing advantage for introgressing *griseorufus*-like alleles. The apparently strong degree of hybridization within the transition zone could thus result from long-term past introgression. Without long-term climate data from this area, it remains unclear if selection in the course of ongoing aridification could be responsible for heterozygote deficiencies and linkage disequilibria within the transition zone. It could even be possible that short-term climatic fluctuations superimpose a long-term trend of aridification and contribute to ongoing change of environmental selective pressures.

### Potential adaptive value of hybridization and maintenance of distinctiveness

The environment-related process proposed by Gligor et al. [30] implies an adaptive value of hybridization in the gradient zone, which facilitates adaptation to changing environmental conditions. In the spiny-gallery zone, so far, we have regarded the apparently great relative abundance of *Microcebus murinus *at Mangatsiaka as a prerequisite that possibly facilitated hybridization. In fact, we were astonished to see that the great majority of individuals at this site carried *murinus-*like haplotypes because it appeared as suboptimal habitat for the species. The headwaters of the temporary watercourses at Mangatsiaka are situated within dry spiny bush to the north and northeast, while those of all other gallery forests in our study are situated in the eastern rainforest. Accordingly, there is only very sparse gallery vegetation at Mangatsiaka. It appears now as possible that *Microcebus murinus *locally adapted to dryer conditions at Mangatsiaka and that hybridization could have facilitated this adaptation.

There is increasing evidence for potentially beneficial consequences of hybridization such as facilitation of adaptability and diversification [2,3,8]. Hybridization can enhance the invasibility of intruding species [74]. For *Microcebus griseorufus *in turn, one could expect rather negative consequences of hybridization. Hybridization with a more common, widespread invader is potentially deleterious and often seen as a threat for rare specialized endemics [4,5]. On the other hand, hybridizing species can remain distinct when hybrid zones are narrow [9] or when genotypes display bimodal distributions [6,75]. Even rare endemic specialists can maintain distinctiveness despite hybridization with a more common invader [76].

*Microcebus griseorufus *and *M. murinus *maintain their distinctiveness in the two contact zones in different ways. In the gradient contact zone, introgressed populations in transitional forest cannot be assigned to either parental species, but the two species remain distinct in adjacent spiny bush and littoral forest. In the spiny-gallery contact zone, the strongly bimodal microsatellite genotypes and their concordance with mitochondrial haplotypes indicate that selection is apparently strong enough to preserve the distinctiveness of both species in the face of local sympatric admixture and bidirectional introgressive hybridization. This does not preclude the potential acquisition of single beneficial adaptations. The identification of respective candidate loci, however, would require a genome wide scan with numerous loci.

## Conclusions

*Microcebus griseorufus *and *M. murinus *are sister species, but they are not among the most closely related species of mouse lemurs detected so far (e.g. [27,29,39]). The fact that they hybridize generates the expectation that hybrid zones between further species of mouse lemurs could exist or could have existed in the past. Our study demonstrates that the two species display very different patterns of hybridization under different ecological conditions. This highlights the importance of environmental factors for the formation of different kinds of contact zones. Our results show that the transition zone of southeastern Madagascar harbors different hybrid zones in very different ecological settings within a small geographic area. This exemplifies that a multitude of opportunities for the formation of different contact and hybrid zones might exist or have existed within the complex biogeography of Madagascar that still need to be explored. Our study points to interesting perspectives on the potential role of hybridization in the evolution of Madagascar's endemics that require further investigation: It appears as possible that macroendemic species acquire beneficial adaptations through hybridization with microendemic congeners that allow them to extend their ranges into novel kinds of habitat. At the same time, the two hybrid zones of mouse lemurs exemplify that species can maintain distinctiveness despite introgressive hybridization.

Madagascar's biogeography has been repeatedly reshuffled during the Pleistocene and Holocene climatic fluctuations. These processes appear as rather recent and rapid when compared to phylogenetic timescales. Consequently, their importance for the diversification of endemics in Madagascar is controversial [15,16,18,24]. It might be possible to reconcile these conflicting views partially when we pay more attention to the rapid evolutionary process of hybridization.

## Authors' contributions

AH supervised all molecular genetic analyses and initial data analyses of a subset of the data, carried out the data analyses with the complete dataset and drafted the manuscript. MG performed a part of the molecular genetic analyses with mitochondrial and nuclear markers. SJR organized and carried out the field study, which was the basis for all genetic and spatial analyses. DR wrote Perl scripts for the analyses of multiple microsatellite datasets and the program SOA, which we used for the analysis of spatial distances between individual capture sites. OZ performed a part of the molecular genetic analyses with mitochondrial and nuclear markers and initial data analyses based on a subset of 77 individual samples. MG, SJR, DR and OZ helped drafting the manuscript by discussions of important parts of the content with AH. All authors read and approved the final manuscript.

## Supplementary Material

Additional file 1 **Individual trap positions at Mangatsiaka and Tsimelahy**. The table contains geographical coordinates of the individual trap positions at Mangatsiaka and Tsimelahy, which we used for analyses of spatial overlap. Click here for file

Additional file 2 **Genotypes**. The table contains the multi-locus genotypes of all individuals. Columns C and D contain the mitochondrial haplotypes and GenBank accession numbers. Column E lists the species-assignment as revealed by tree reconstructions based on mitochondrial haplotypes. Columns F to N contain the microsatellite genotypes in Genepop two-digit format. Click here for file

Additional file 3 **Proportion of *Microcebus murinus *within the nearest neighborhood of *M. griseorufus***. The figure displays the average proportion of individuals with *murinus-*like mitochondrial haplotypes within different numbers of nearest neighbors to individuals with *griseorufus-*like haplotypes. We used three datasets: Mtk: Mangatsiaka; Mtk_Riv: Mangatsiaka, all *murinus-*like individuals sampled more than 50 m from the nearest watercourse excluded; Tml: Tsimelahy. For each dataset, we calculated the proportion based on all individuals (_all) and as an average over 100 randomly resampled datasets, where we reduced the number of *murinus-*like individuals to the number of *griseorufus-*like individuals (_res). Click here for file

Additional file 4 **Diameters of neighborhoods with different numbers of nearest neighbors**. The figure displays the average diameters of the nearest neighborhoods of individuals with *griseorufus-*like mitochondrial haplotypes at Mangatsiaka and Tsimelahy. We calculated average diameters for neighborhoods including different numbers of nearest neighbors based on three datasets: Mtk: Mangatsiaka; Mtk_Riv: Mangatsiaka, all *murinus-*like individuals sampled more than 50 m from the nearest watercourse excluded; Tml: Tsimelahy. For each dataset, we calculated diameters based on all individuals (_all) and as an average over 100 randomly resampled datasets, where we reduced the number of *murinus-*like individuals to the number of *griseorufus-*like individuals (_res). Click here for file

Additional file 5 **Simulation A, STRUCTURE, observed proportions with a threshold of 0.1**. The table displays the results of simulation A with STRUCTURE with a threshold value for hybrid detection of 0.1. Click here for file

Additional file 6 **Simulation B: scenario of no hybrids at Mangatsiaka**. The table displays the results of simulation B. Click here for file

Additional file 7 **Re-evaluated identification of hybrids in the gradient contact zone**. The figure displays some of the original results of Gligor et al. [30] from the gradient contact zone, which we re-evaluated under application of the same criteria for hybrid detection as in the study presented here. Upper row: mitochondrial data (mt), middle and lower row membership coefficients observed with STRUCTURE with K = 2 in a large and a smaller dataset. Each vertical bar represents one individual. Colors represent the two species. Mv, Be, Amp, Sak, Ank, Anj, Pe, Man, Lok: abbreviations of sampling sites. Sampling sites are aligned in west-eastern direction along the transect sampled by Gligor et al. [30]. Gligor et al. [30] present the exact localities and full names of these sites in their Figure 1 and Table 1. Click here for file
