# Supplementary figures and images for: Hybridization of mouse lemurs: different patterns under different ecological conditions
Source: BMC Evol Biol. 2011 Oct 11;11:297. doi: 10.1186/1471-2148-11-297 (PMC3206491; doi:10.1186/1471-2148-11-297)

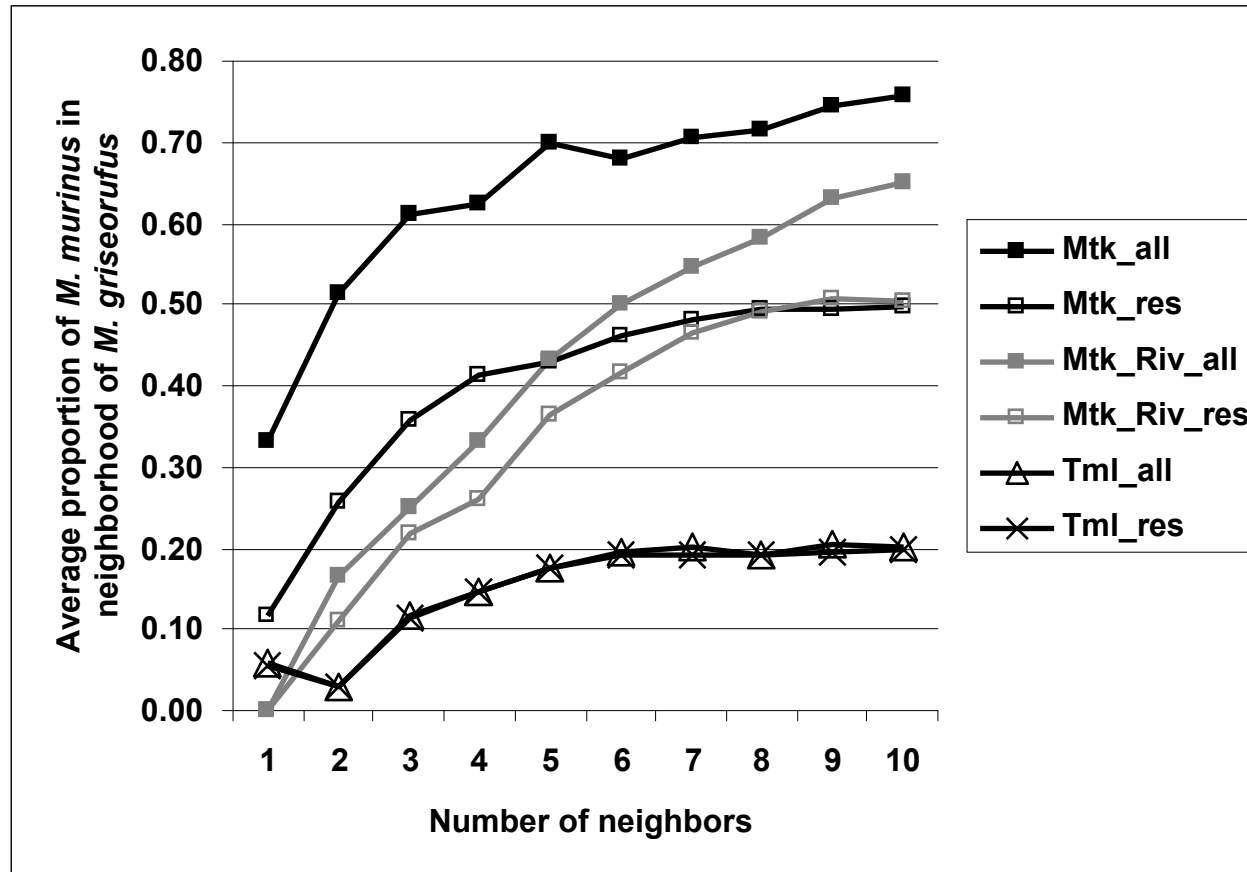

Supplement: Additional file 3 — Proportion of Microcebus murinus within the nearest neighborhood of M. griseorufus. The figure displays the average proportion of individuals with murinus-like mitochondrial haplotypes within different numbers of nearest neighbors to individuals with griseorufus-like haplotypes. We used three datasets: Mtk: Mangatsiaka; Mtk_Riv: Mangatsiaka, all murinus-like individuals sampled more than 50 m from the nearest watercourse excluded; Tml: Tsimelahy. For each dataset, we calculated the proportion based on all individuals (_all) and as an average over 100 randomly resampled datasets, where we reduced the number of murinus-like individuals to the number of griseorufus-like individuals (_res). [file 1471-2148-11-297-S3.PDF]

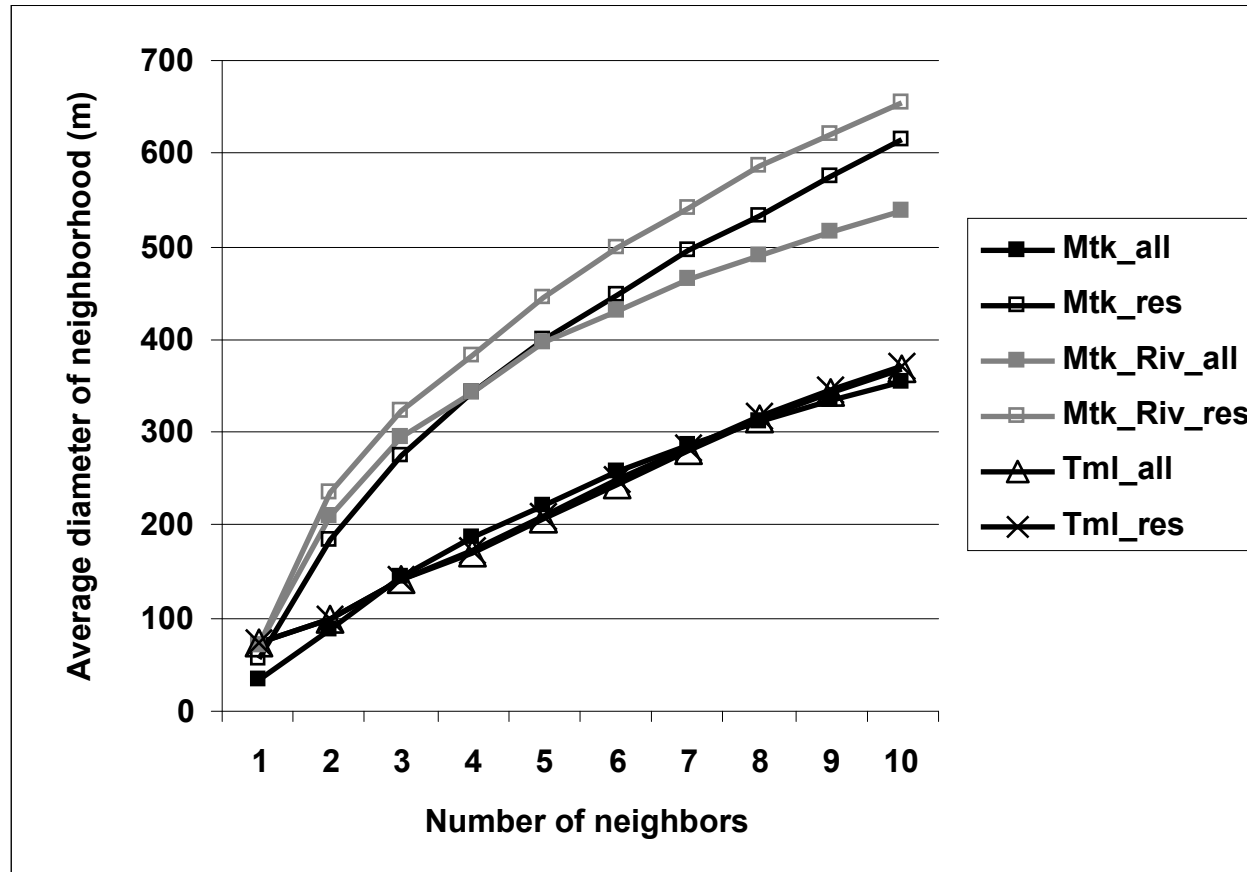

Supplement: Additional file 4 — Diameters of neighborhoods with different numbers of nearest neighbors. The figure displays the average diameters of the nearest neighborhoods of individuals with griseorufus-like mitochondrial haplotypes at Mangatsiaka and Tsimelahy. We calculated average diameters for neighborhoods including different numbers of nearest neighbors based on three datasets: Mtk: Mangatsiaka; Mtk_Riv: Mangatsiaka, all murinus-like individuals sampled more than 50 m from the nearest watercourse excluded; Tml: Tsimelahy. For each dataset, we calculated diameters based on all individuals (_all) and as an average over 100 randomly resampled datasets, where we reduced the number of murinus-like individuals to the number of griseorufus-like individuals (_res). [file 1471-2148-11-297-S4.PDF]

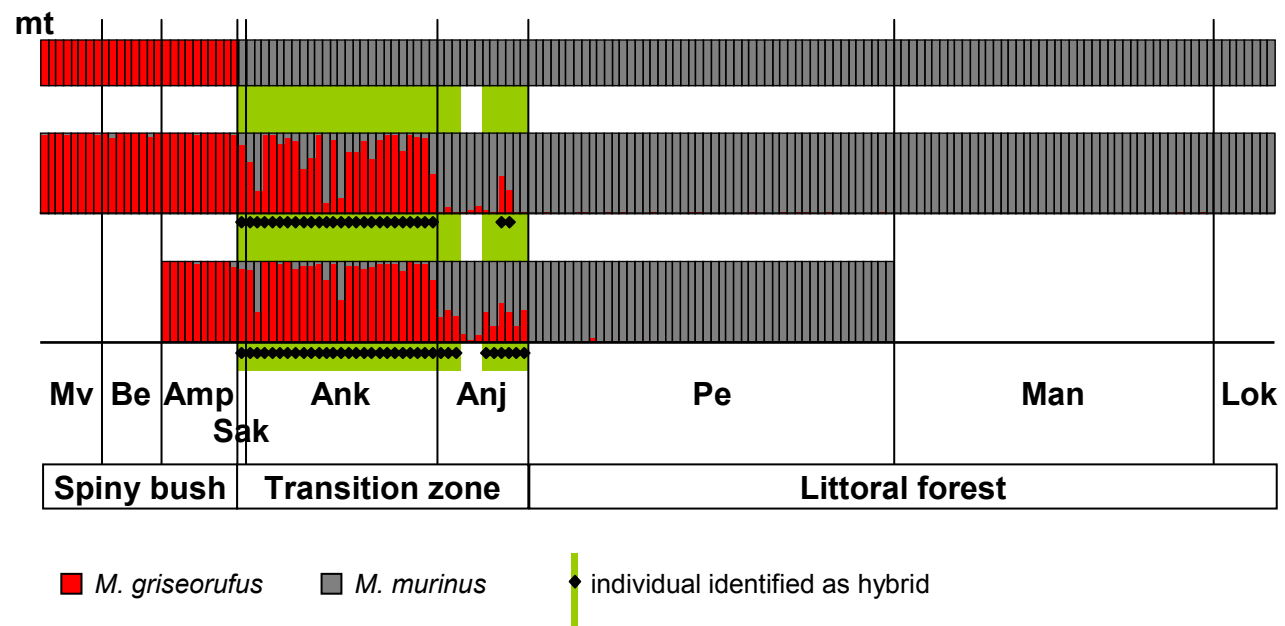

Supplement: Additional file 7 — Re-evaluated identification of hybrids in the gradient contact zone. The figure displays some of the original results of Gligor et al. [30] from the gradient contact zone, which we re-evaluated under application of the same criteria for hybrid detection as in the study presented here. Upper row: mitochondrial data (mt), middle and lower row membership coefficients observed with STRUCTURE with K = 2 in a large and a smaller dataset. Each vertical bar represents one individual. Colors represent the two species. Mv, Be, Amp, Sak, Ank, Anj, Pe, Man, Lok: abbreviations of sampling sites. Sampling sites are aligned in west-eastern direction along the transect sampled by Gligor et al. [30]. Gligor et al. [30] present the exact localities and full names of these sites in their Figure 1 and Table 1. [file 1471-2148-11-297-S7.PDF]
