# Supplementary material for: Hybridization of mouse lemurs: different patterns under different ecological conditions
Source: BMC Evol Biol. 2011 Oct 11;11:297. doi: 10.1186/1471-2148-11-297 (PMC3206491; doi:10.1186/1471-2148-11-297)
Supplement: Additional file 5 — Simulation A, STRUCTURE, observed proportions with a threshold of 0.1. The table displays the results of simulation A with STRUCTURE with a threshold value for hybrid detection of 0.1. [file 1471-2148-11-297-S5.PDF]

Simulation A, STRUCTURE, observed proportions with a threshold of 0.1

	Mg	Mm	Hyb
Mg (n=10,000)	1.000	0.000	0.000
Mm (n=10,000)	0.000	0.998	0.002
F1 (n=1000)	0.014	0.006	0.980
F2 (n=1000)	0.044	0.033	0.923
Mg-Bx1 (n=1000)	0.461	0.002	0.537
Mm-Bx1 (n=1000)	0.001	0.475	0.524
Mg-Bx2 (n=1000)	0.826	0.000	0.174
Mm-Bx2 (n=1000)	0.000	0.815	0.185

The table displays the proportions of simulated individuals of different categories assigned to different categories under a threshold value of 0.1, which we also applied to the real data. Columns : observed categories, rows: true categories; Mg: purebred *Microcebus griseorufus*, Mm: purebred *M. murinus*, F1: Mg x Mm, F2: F1 x F1, Mg-Bx1: F1 x Mg, Mm-Bx1: F1 x Mm, Mg-Bx2: Mg-Bx1 x Mg, Mm-Bx2: Mm-Bx1 x Mm, Hyb: hybrid.
