# Supplementary material for: Hybridization of mouse lemurs: different patterns under different ecological conditions
Source: BMC Evol Biol. 2011 Oct 11;11:297. doi: 10.1186/1471-2148-11-297 (PMC3206491; doi:10.1186/1471-2148-11-297)
Supplement: Additional file 6 — Simulation B: scenario of no hybrids at Mangatsiaka. The table displays the results of simulation B. [file 1471-2148-11-297-S6.PDF]

Simulation B: scenario of no hybrids at Mangatsiaka

	M. griseorufus	*M. murinus*
Simulated purebred individuals	1200	6300
STRUCTURE: correctly identified	100%	99.46%
STRUCTURE: Prop. sets (nHyb ≥ real)	NA	0.00
NEWHYBRIDS: correctly identified	94.67%	99.94%
NEWHYBRIDS: Prop. sets (nHyb ≥ real)	0.04	0.00

The table displays the results from 100 simulated datasets with each 12 purebred *Microcebus griseorufus* and 63 purebred *M. murinus*. Correctly identified: percentage of individuals identified as purebred, Prop. sets (nHyb ≥ real): proportion of simulated datasets with an observed number of hybrids equal to or greater than in the real data. In the real data, STRUCTURE had identified 7 hybrids with *murinus*-like mitochondrial haplotypes. NEWHYBRIDS had identified 3 hybrids with *griseorufus*-like and 6 with *murinus*-like haplotypes. NA: not applicable since STRUCTURE detected no hybrids in the real data.
